# Increased tibiofemoral rotation is associated with anatomical risk factors for lateral patella instability

**DOI:** 10.1002/jeo2.70626

**Published:** 2026-01-09

**Authors:** Julius Watrinet, Lennart Gerdesmeyer, Felix Meurer, Romed P. Vieider, Armin Runer, Sebastian Siebenlist, Lukas Willinger

**Affiliations:** ^1^ Department of Orthopaedic Sports Medicine TUM University Hospital Ismaninger Munich Germany; ^2^ Department of Diagnostic and Interventional Radiology TUM University Hospital Ismaninger Munich Germany

**Keywords:** deformity, instability, patella, risk factor

## Abstract

**Purpose:**

Chronic patellofemoral instability (PFI) and pain are influenced by axial malalignment of the patellofemoral joint. Tibiofemoral rotation, defined as the rotation between femur and tibia knee, has shown to be correlated to PFI. This study aimed to determine whether tibiofemoral rotation is associated with anatomical risk factors for patellofemoral maltracking in patients with PFI. It was hypothesised that greater tibiofemoral rotation correlates with other predisposing factors for PFI.

**Methods:**

Eighty‐five consecutive patients (mean age 22.6 ± 8.9 years; 58 female) with PFI underwent standardised bilateral knee MRI in 0° extension for rotational analysis. Tibiofemoral rotation was measured as the angle between the posterior femoral condylar tangent and posterior tibial plateau tangent on axial images. Ipsilateral and contralateral values along other anatomical patellofemoral risk factors were recorded. One‐sample *t‐*tests compared to the healthy contralateral side. Pearson correlations assessed associations between tibiofemoral rotation and anatomic risk factors for patellofemoral maltracking.

**Results:**

Mean ipsilateral tibiofemoral rotation was 6.9° ± 6.0° (range: −6.5° to 21.6°). Contralateral version averaged 6.4° ± 6.2° with no significant side difference. Absolute side‐to‐side difference was 3.7° ± 2.7°. Ipsilateral tibiofemoral rotation correlated with the tuberositas tibiae–trochlea groove (TT–TG) and medial posterior cruciate ligament distance (TT–PCL) (*r* = 0.37, *p* < 0.001, *r* = 0.43, *p* < 0.001), femoral torsion (*r* = −0.32, *p* = 0.003) and lateral trochlea inclination (*r *= −0.344, *p* = 0.001) whereas other patellofemoral risk factors showed no correlation.

**Conclusion:**

Tibiofemoral rotation showed a significant correlation with anatomical risk factors associated with lateral patellar maltracking in patients with PFI. Moreover, patients with PFI demonstrated a wide variability in tibiofemoral rotation. Consideration of tibiofemoral rotation may enhance anatomical assessment and aid clinical decision‐making in patients with PFI.

**Level of Evidence:**

Level III.

**Abbreviations**
CI: caton–deschamps index
HKA: hip–knee–ankle angle
ICC: intraclass correlation coefficient
MRI: magnetic resonance imaging
PCL: posterior cruciate ligament
PFI: patellofemoral Instability
Q‐Angle: quadriceps angle
SD: standard deviation
TTO: tibial tubercle osteotomy
TT–PCL: tibial tubercle–posterior cruciate ligament distance
TT–TG: tibial tubercle–trochlear groove distance

## INTRODUCTION

Chronic patellofemoral instability (PFI) is a multifactorial disorder characterised by recurrent lateral patellar displacement, pain, functional impairment and cartilage degeneration [10, 12, 24]. Recognised anatomical risk factors include trochlear dysplasia, patella alta, valgus malalignment, increased tibial tubercle–trochlear groove (TT–TG) distance and pathologic femoral or tibial torsion [3, 6, 10, 13, 14, 17, 23].

Tibiofemoral rotation, defined as the relative rotation between the distal femur and proximal tibia in 0° extension, is measured on axial imaging as the angle between the posterior femoral condylar tangent and the posterior tibial plateau tangent [16]. Originally described via CT, tibiofemoral rotation measurement demonstrates excellent reliability (ICC ≥ 0.90) on magnetic resonance imaging (MRI) [12, 16]. Huettner et al. established healthy norms in 100 asymptomatic volunteers (mean 27.5 years; 55% female), reporting a mean external tibiofemoral rotation of 1.3° ± 3.9°.

Recent studies suggest a link between elevated tibiofemoral rotation and patellofemoral disorders. Significantly higher external tibiofemoral rotation was found in patients with anterior knee pain [12]. Furthermore, increased tibiofemoral rotation is associated with patellar dislocations, independent of trochlear morphology [11]. Biomechanically, lateralisation of the tibial tubercle due to external tibiofemoral rotation alters patellar tracking by increasing the dynamic Q‐angle and lateral shear forces [25].

Therefore, this study aimed to determine whether tibiofemoral rotation is higher in patients with chronic PFI compared to the healthy contralateral knee, as well as to assess its correlation with anatomical risk factors for patellofemoral maltracking and pain. It was hypothesised that patients would exhibit significantly higher external tibiofemoral rotation than the normative mean and that tibiofemoral rotation would correlate positively with TT–TG distance.

## MATERIALS AND METHODS

Eighty‐five patients aged 11–46 years (mean 22.6 ± 8.9; 58 female) who presented with chronic PFI who underwent in‐house MRI rotational analysis between February 2022 and November 2024 were included. Chronic PFI was defined as a history of at least one documented patellar dislocation combined with a persistent subjective feeling of instability for more than 3 months despite conservative treatment. Exclusion criteria included incomplete or inadequate imaging and prior arthroplasty or osteotomy. Eighty‐five out of 100 screened patients met inclusion criteria.

**Figure 1:**
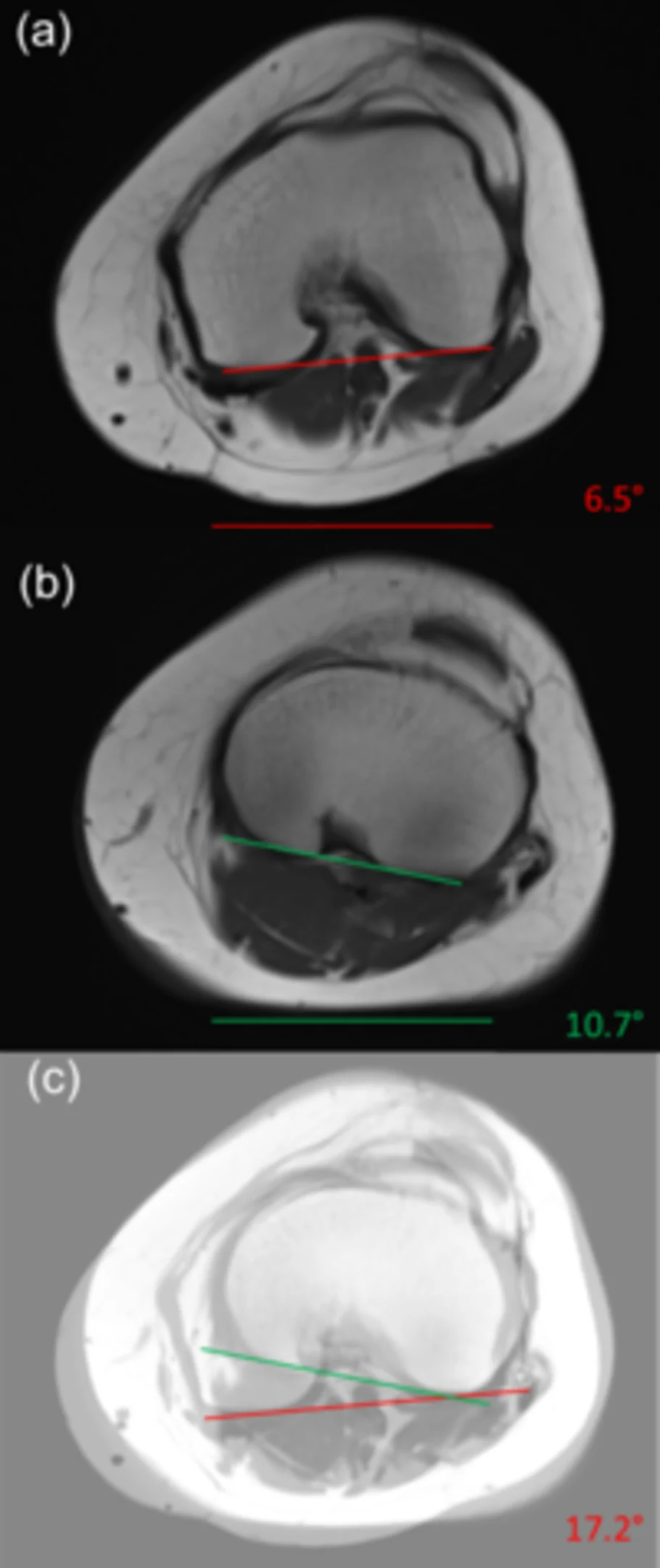
Demonstration of the measurement of the tibiofemoral rotation according to Eckhoff et al. [12]. Tibiofemoral rotation was assessed using axial T2‐weighted MR images. (a) At the distal femur, a line (red) was drawn connecting the posterior aspects of the femoral condyles. (b) At the proximal tibia, a line (green) was drawn along the posterior cortical margin of the tibial plateau. (c) The angle between the femoral and tibial lines results from overlaying a and b and represents the tibiofemoral rotation, calculated as the angle between the posterior femoral condylar axis and the posterior tibial axis.

Bilateral MRIs of the hip, knee and ankle were obtained with the limb in 0° extension and neutral hip and ankle position in patients presenting with clinical signs of rotational malalignment. Tibiofemoral rotation was measured at the level of maximal femoral condylar width as the angle between the posterior femoral condylar tangent and posterior tibial plateau tangent (Figure 1; inter‐rater reliability 0.93) [16]. Femoral torsion was assessed via the Waidelich method; tibial torsion was measured from the posterior tibial plateau to the bimalleolar axis [24]. On ipsilateral MRI scans, TT–TG [14] and tibial tubercle–posterior cruciate ligament distance (TT–PCL) [23] distances were determined on axial images as the mediolateral offset between tibial tubercle and trochlear groove and the tibial insertion of the posterior cruciate ligament. Patellar height was evaluated using the caton–deschamps index on sagittal radiographs [6]. The Biedert‐Index, which quantifies the cartilaginous congruence of the patellofemoral joint, was calculated on sagittal MRI as the percentage of patellar articular cartilage length overlapped by trochlear cartilage [3]. Lateral trochlea inclination was determined according to Carillon et al. [5]. The hip‐knee‐ankle angle was measured on standardised full‐leg weight‐bearing antero‐posterior radiographs [8]. Dysplasia of the trochlea was graded according to Dejour et al. [9]. For classification, normative tibiofemoral rotation was defined as values within one standard deviation of the mean reported by Huettner et al. This retrospective cohort study was approved by the Institutional Review Board (IRB No. 2022‐223‐S‐NP).

### Statistical analysis

Continuous variables are reported as mean ± SD; categorical as counts (percentages). Normality was confirmed by Shapiro–Wilk tests. Paired *t*‐tests assessed side‐to‐side differences. Pearson correlation coefficients evaluated associations between tibiofemoral rotation and TT–TG, femoral torsion and caton–deschamps index. Pearson correlation coefficients (*r*) were interpreted according to established conventions, with *r* < 0.3 considered low, 0.3–0.5 moderate and > 0.5 high correlation, as described by Cohen et al. [7]. Group differences were evaluated using Fisher′s exact test as appropriate. An a priori power analysis was performed using G*Power (version 3.1.9.6) for a two‐tailed independent‐samples *t*‐test with *α* = 0.05 and power of 80%. Based on mean values for tibiofemoral rotation reported for healthy individuals (group 1: 1.3° ± 7.2°) and patients with patellofemoral pain (group 2: 3.9° ± 1.1°), the calculated effect size was *d* = 0.50, yielding a required sample size of *n* = 63. Significance was set at *p* < 0.05. Analyses were performed in r Studio (Version 2023.03.1 + 446). ChatGPT‐5 (OpenAI) was employed for language editing of this manuscript.

## RESULTS

Patient axial alignment parameters are summarised in Table 1. Mean ipsilateral tibiofemoral rotation was 6.9° ± 6.0°, ranging from −6.5° to 21.6°. Contralateral tibiofemoral rotation averaged 6.4 ± 6.2°. The mean side‐to‐side difference (3.7° ± 2.7) was not significant (*p* = 0.28).

**Table 1:** Axial alignment parameters—side‐to‐side comparison (paired *t*‐test).

	Ipsilateral	Contralateral	*p* value
Tibiofemoral rotation	6.9 ± 6.0	6.4 ± 6.2	0.284
Femoral torsion	−17.1 ± −11.0	−16.0 ± −12.4	0.194
Tibial torsion	34.2 ± 7.9	34.5 ± 8.6	0.501

**Figure 2:**
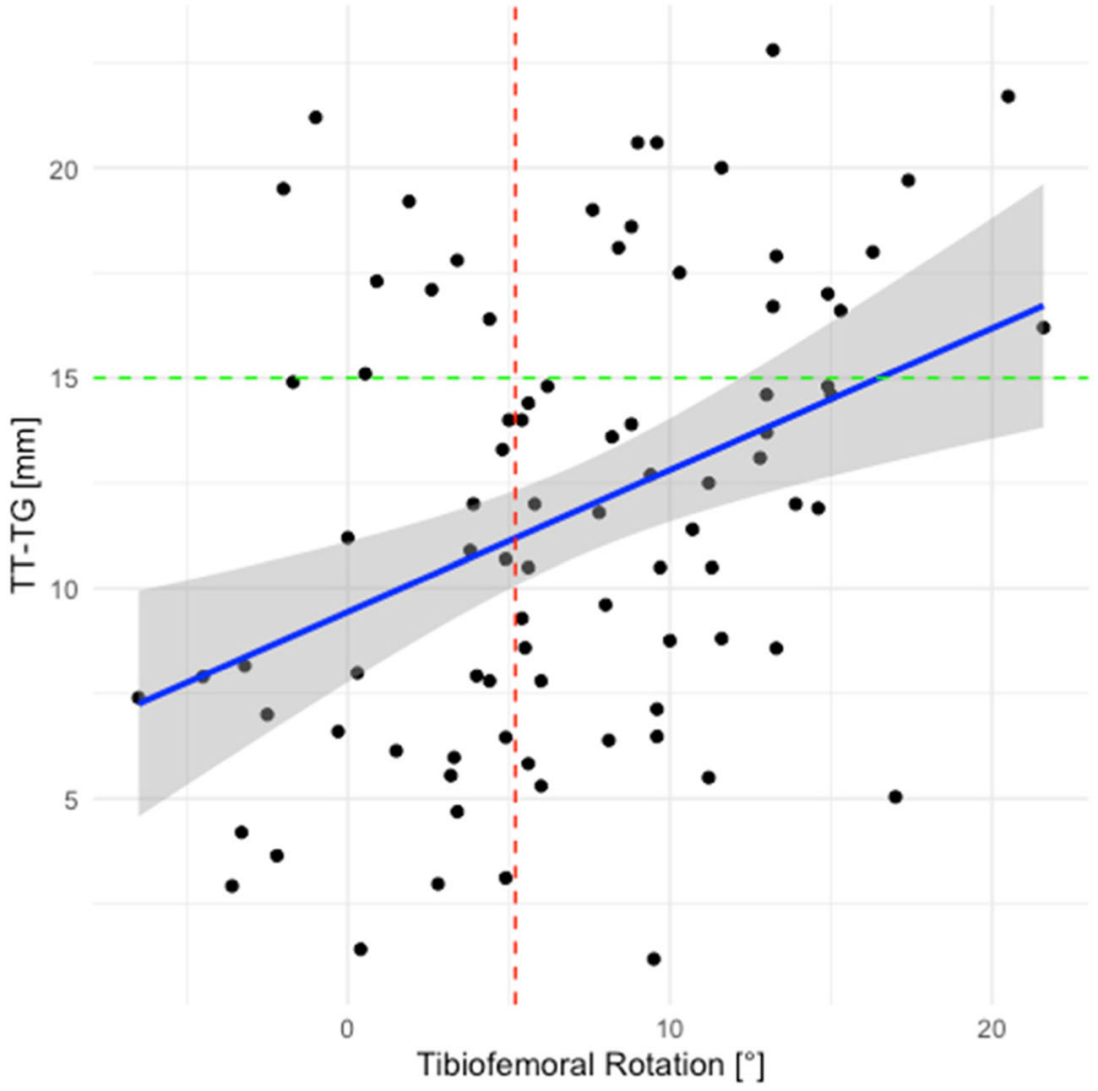
Scatterplot of ipsilateral tibiofemoral rotation (°) versus TT–TG distance (mm). The regression line denotes *r* = 0.37, *p* < 0.001.

**Figure 3:**
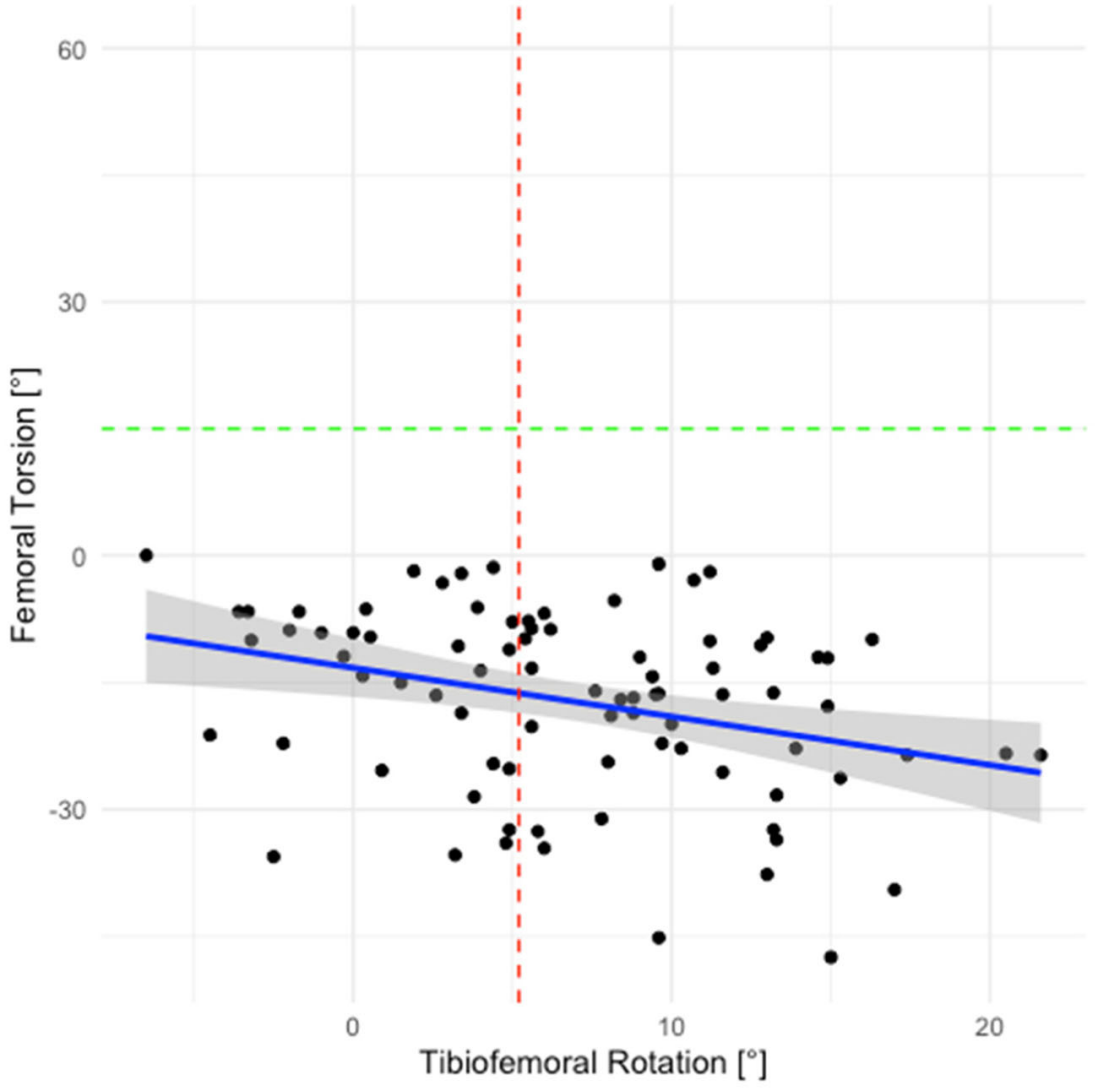
Scatterplot of ipsilateral tibiofemoral rotation (°) versus femoral torsion (°). The regression line denotes *r* = − 0.32, *p* = 0.003.

**Figure 4:**
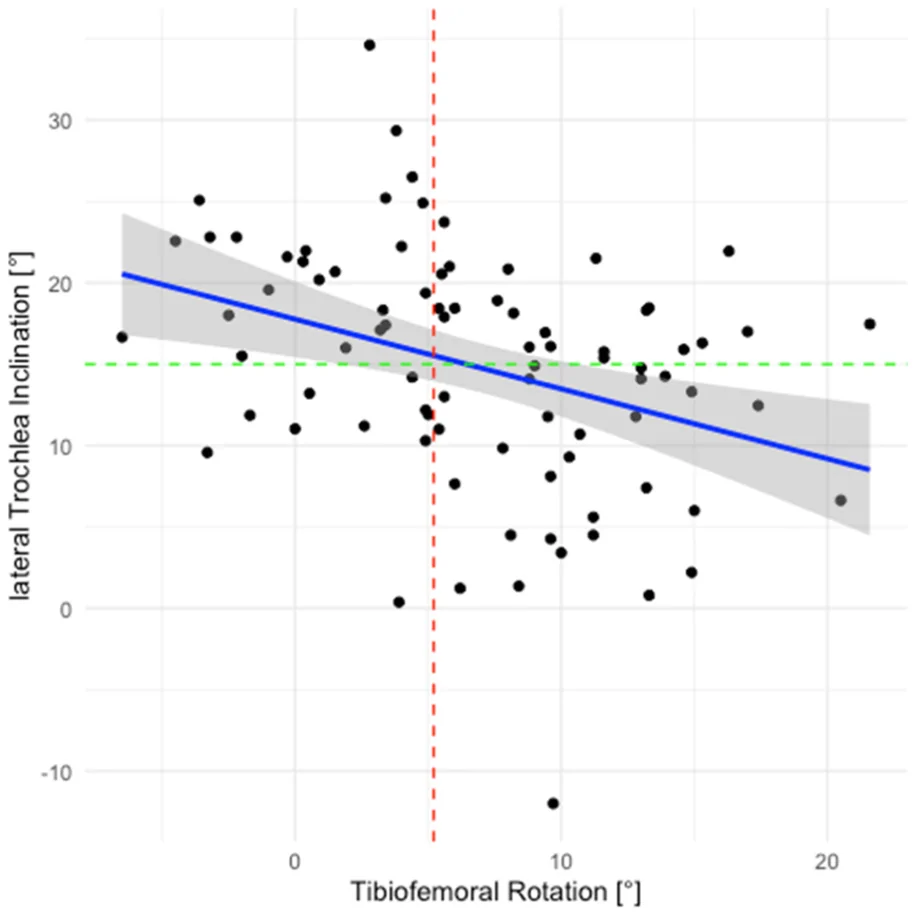
Scatterplot of ipsilateral tibiofemoral rotation (°) versus lateral trochlea inclination (°). The regression line denotes *r* = −0.344, *p* = 0.001.

Regarding risk factors for PFI, ipsilateral tibiofemoral rotation correlated moderately with TT–TG distance (*r* = 0.37, *p* = 0.004) and negatively with femoral torsion (−0.35, *p* = 0.001) (Tables 2 and 3). In 32% (*n* = 27) of cases, the tibiofemoral rotation was elevated while the TT–TG was in a normal range (Table 4). Figures 2, 3, 4 illustrate the scatterplot of tibiofemoral rotation versus TT–TG distance, femoral torsion and lateral trochlea inclination.

**Table 2:** Patellofemoral risk factors and their correlation with the ipsilateral tibiofemoral rotation.

Variable	Mean ± SD or *n* (%)	Correlation with tibiofemoral rotation
TT–TG distance, mm	11.8 ± 5.4	*r* = 0.37, *p* = 0.004
TT–PCL distance, mm	19.7 ± 5.5	*r* = 0.43, *p* < 0.001
Caton–deschamps index	1.2 ± 0.2	*p* = 0.959
Hip‐knee‐ankle‐angle, degrees	180.8 ± 3.3	*p* = 0.541
Q angle, degrees (*n* = 65)	4.9 ± 7.9	*p* = 0.990
Biedert index, percentage	40 ± 16	*p* = 0.777
Lateral Trochlea inclination, degrees	14.8 ± 7.5	*r* = −0.35, *p* = 0.001

Abbreviations: TT–PCL, tibial tubercle–posterior cruciate ligament distance; TT–TG, tibial tubercle–trochlear groove.

**Table 3:** Dysplasia of the Trochlea was graded according to Dejour [5].

No dysplasia	A	B	C	D
4	19	20	39	3

**Table 4:** Association between tibiofemoral rotation and TT–TG categories.^a^

		Tuberositas tibiae–trochlea groove distances (TT–TG)		
		Normal (≤15 mm)	Borderline (15–20 mm)	Pathologic (>20 mm)
Tibiofemoral rotation	Norm	34 (40%)	0 (0%)	0 (0%)
	Elevated	27 (32%)	18 (21%)	6 (7%)

^a^ Cross‐tabulation of tibiofemoral rotation status (normal [< 5.2°] vs. elevated [>5.2]) and tibial tuberosity–trochlear groove (TT–TG) categories. TT–TG values were stratified into normal (≤15 mm), borderline (15–20 mm) and pathological (>20 mm). Absolute counts and total percentages are provided.

## DISCUSSION

The most important finding of this study was that patients with chronic PFI exhibit a tibiofemoral rotation of 6.9° ± 6.0° using the measurement technique described by Eckhoff et al. [12]. The reported tibiofemoral rotation values exceed the reference of 1.3° ± 3.9° for healthy individuals [16]. The wide range observed highlights considerable interindividual variability and suggests that tibiofemoral rotation represents one of several contributing factors in PFI. Tibiofemoral rotation showed positive correlations with the TT–TG and TT–PCL distances and negative correlations with femoral torsion and lateral trochlear inclination. Although the correlations were only of moderate strength, they indicate interrelations among anatomical risk factors contributing for PFI. Other patellofemoral risk factors showed no significant associations.

Because of the influence of the screw‐home mechanism, which is described as an outward rotation of the tibia during the final extension, values should be compared only at equivalent positions of extension or flexion [1, 19]. Given the differences of 5.6° between normal and pathological tibiofemoral rotation reported in the literature, variations between imaging modalities appear relevant and may critically influence clinical interpretation.

The findings of the current study were therefore compared only with values of studies reporting tibiofemoral rotation in 0° extension. A mean value of 7.2° ± 1.1° for patients with anterior knee pain was reported [12]. Similar results were reported, with tibiofemoral rotation measuring 8.2° ± 6.5° in patients with PFI [1]. In a comparative study, a mean tibiofemoral rotation of 8.8° was reported in patients with PFI compared to 3.8° in healthy controls [20]. In paediatric and adolescent patients with PFI, an increased tibiofemoral rotation (6.9°) was demonstrated compared to individuals without instability (0.8). Those values were slightly smaller compared to the above‐mentioned results, as all measurements were obtained in standard MRI scans with the knee in slight flexion (20°) [2]. Another study assessing knees in standard MRIs by Lin et al. observed a graded increase based on severity, ranging from 8.5° in severe cases to −3.8° in healthy controls [22]. As values up to 5.7° have been reported in healthy cohorts, the definition of a pathological threshold remains challenging [11].

With respect to torsional alignment, the standard value for femoral torsion is −20.4° ± 9° and for tibial torsion 33.1° ± 8° defined by Waidelich et al. [24]. The measured values in the present cohort, therefore, suggest that, on average, no isolated axial deformity of the femur or tibia was present, which might have influenced the tibiofemoral rotation.

The observed correlation between tibiofemoral rotation and TT–TG distance (*r* = 0.42) underscores how tibiofemoral rotation contributes to the lateralisation of the tibial tubercle. Lateral displacement of the tibial tubercle increases TT–TG distance and predisposes to lateral patellar tracking. Increased tibiofemoral rotation, which corresponds to an externally rotated tibia relative to the femur, also increases the functional Q‐angle. This geometric alteration shifts the line of pull of the quadriceps laterally, augments the valgus vector acting on the patella and thereby favours lateral tracking and potential dislocation. Notably, in approximately one third of patients tibiofemoral rotation was increased despite the TT–TG distance remaining within the normative range (<15 mm, Table 4), highlighting that pathological cut‐offs for tibiofemoral rotation have not yet been defined and that clinically relevant malalignment may be overlooked if TT–TG is considered in isolation [4, 26].

Preoperatively, torsional assessment should extend beyond isolated femoral and tibial measures to include tibiofemoral rotation. Restoration of native tibiofemoral rotation in total knee arthroplasty has been shown to improve patellar alignment, indicating surgical relevance [18]. However, to date, literature is scarce on surgical techniques that solely correct tibiofemoral rotation. Derotational osteotomies targeting the cumulative rotational mismatch may optimise patellar tracking, but did not show to influence tibiofemoral rotation postoperatively [21]. Although tibiofemoral rotation and the TT–TG distance correlate positively, evidence regarding the effect of tibial tubercle osteotomy with medialization on tibiofemoral rotation is conflicting: while one study reported no postoperative change, another demonstrated a reduction in tibiofemoral rotation following the procedure [15, 27]. A positive effect of tibial tubercle osteotomy on tibiofemoral rotation might be due to an alteration of the Q‐angle [25].

Although in the present study only symptomatic knees were analysed, side‑to‑side comparison revealed no significant difference in tibiofemoral rotation between the affected and contralateral healthy extremity. This symmetry suggests that the rotational morphology is predominantly developmental and not acquired through recurrent dislocations or adaptive changes. Accordingly, the contralateral knee may exhibit a comparable constellation of risk factors and thus be predisposed to future PFI, even if currently asymptomatic. Bilateral manifestations of torsional abnormalities have been documented in previous studies, highlighting the need for thorough patient counselling and, when appropriate, systematic evaluation of the contralateral limb.

The main limitation of this study is the incomplete understanding of the underlying anatomical and functional contributors to tibiofemoral rotation. It remains unclear to what extent soft tissue contributes to the tibiofemoral rotations since the measured values primarily reflect the rotation between the femur and tibia. The bilateral MRI was conducted under static, nonweight‐bearing conditions, which may not fully reflect dynamic knee mechanics—especially the screw‐home mechanism. Only patients who underwent comprehensive rotational MRI were included, introducing a potential selection bias as individuals with similar symptoms who did not receive full rotational imaging were not captured and may limit the generalisability of the findings. Additionally, the absence of a healthy control group limits comparability, although normative values were suggested in the literature. Finally, the single‐centre sample may affect generalizability.

The results show the multifactorial and interdependent nature of chronic PFI. The tibiofemoral rotation seems to contribute to a more lateral patella position and should be measured in these patients as well as incorporated in the treatment considerations. Its correlation with the TT–TG and femoral torsion may offer surgical options like tuberositas tibiae medialization in the future. However, more studies need to investigate the effect of these procedures on tibiofemoral rotation.

## CONCLUSION

Tibiofemoral rotation shows a significant correlation with anatomical risk factors associated with lateral patellar maltracking in patients with PFI. Moreover, patients with chronic PFI demonstrated a wide variability in tibiofemoral rotation. Consideration of tibiofemoral rotation may enhance anatomical assessment and aid clinical decision‐making in patients with PFI.

## AUTHOR CONTRIBUTIONS

Julius Watrinet and Lukas Willinger designed the study. Lennart Gerdesmeyer collected data. Julius Watrinet and Romed P. Vieider performed the statistical analysis. Julius Watrinet, Lukas Willinger and Armin Runer wrote the manuscript. Felix Meurer and Sebastian Siebenlist assisted with data interpretation and critically reviewed the manuscript. All authors read and approved the final manuscript.

## CONFLICT OF INTEREST STATEMENT

One or more of the authors has declared a potential conflict of interest. Lennart Gerdesmeyer, Romed P. Vieider and Armin Runer report no conflict of interest. Julius Watrinet is a consultant for KLS Martin Group. Sebastian Siebenlist is a consultant for Arthrex GmbH, KLS Martin Group and medi GmbH & Co. KG.

## ETHICS STATEMENT

Ethical approval was given by the local ethics committee of the Technical University Munich (2022‐223‐S‐NP). This is a retrospective study. All patient information was deidentified, and patient consent was not required.

## Data Availability

The data that support the findings of this study are available from the corresponding author upon reasonable request.
